# Digitalization of routine health information systems: Bangladesh, Indonesia, Pakistan

**DOI:** 10.2471/BLT.22.287816

**Published:** 2022-08-22

**Authors:** Tigest Tamrat, Subhash Chandir, Kelsey Alland, Alisa Pedrana, Mubarak Taighoon Shah, Carolyn Footitt, Jennifer Snyder, Natschja Ratanaprayul, Danya Arif Siddiqi, Numera Nazneen, Inraini Fitria Syah, Roger Wong, Peter Lubell-Doughtie, Annisa Dwi Utami, Khaerul Anwar, Hasmot Ali, Alain B Labrique, Lale Say, Anuraj H Shankar, Garrett Livingston Mehl

**Affiliations:** a UNDP/UNFPA/UNICEF/World Bank Special Programme of Research, Development and Research Training in Human Reproduction (HRP), Department of Sexual and Reproductive Health and Research, World Health Organization, Avenue Appia 20, 1211 Geneva 27, Switzerland.; b IRD Global, Singapore, Singapore.; c Department of International Health, Johns Hopkins Bloomberg School of Public Health, Baltimore, United States of America (USA).; d Summit Institute for Development, Mataram, Indonesia.; e Interactive Research and Development Karachi, Pakistan.; f Ona, Nairobi, Kenya.; g Jhpiego, Baltimore, USA.; h Department of Digital Health and Innovations, World Health Organization, Geneva, Switzerland.; i mPower Social Enterprises Ltd., Dhaka, Bangladesh.; j Institute for Health Worker Training of West Nusa Tenggara Province, Mataram, Indonesia.; k JiVitA Maternal and Child Health & Nutrition Research Project, Rangpur, Bangladesh.; l Oxford University Clinical Research Unit–Indonesia, University of Oxford, Oxford, England.; Correspondence to Tigest Tamrat (email: tamratt@who.int).

## Abstract

**Objective:**

To describe a systematic process of transforming paper registers into a digital system optimized to enhance service provision and fulfil reporting requirements.

**Methods:**

We designed a formative study around primary health workers providing reproductive, maternal, newborn and child health services in three countries in Bangladesh, Indonesia and Pakistan. The study ran from November 2014 to June 2018. We developed a prototype digital application after conducting a needs assessment of health workers’ responsibilities, workflows, routine data requirements and service delivery needs. Methods included desk reviews, focus group discussions, in-depth interviews; data mapping of paper registers; observations of health workers; co-design workshops with health workers; and usability testing. Finally, we conducted an observational feasibility assessment to monitor uptake of the application.

**Findings:**

Researchers reviewed a total of 17 paper registers across the sites, which we transformed into seven modules within a digital application running on mobile devices. Modules corresponded to the services provided, including household enumeration, antenatal care, family planning, immunization, nutrition and child health. A total of 65 health workers used the modules during the feasibility assessment, and average weekly form submissions ranged from 8 to 234, depending on the health worker and their responsibilities. We also observed variability in the use of modules, requiring consistent monitoring support for health workers.

**Conclusion:**

Lessons learnt from this study shaped key global initiatives and resulted in a software global good. The deployment of digital systems requires well-designed applications, change management and strengthening human resources to realize and sustain health system gains.

## Résumé

**Objectif:**

Décrire un processus méthodique de transformation des registres papier en système numérique optimisé, en vue d'améliorer la fourniture de services et de remplir les exigences relatives à l'établissement de rapports.

**Méthodes:**

Nous avons conçu une étude formative consacrée aux professionnels des soins primaires proposant des services de santé reproductive et maternelle, de santé des nouveau-nés et de santé infantile dans trois pays: le Bangladesh, l'Indonésie et le Pakistan. Cette étude a été menée entre novembre 2014 et juin 2018. Nous avons mis au point un prototype d'application numérique après avoir évalué les besoins des soignants, leurs responsabilités, leur charge de travail, les données nécessaires à leurs activités quotidiennes et les impératifs liés à leurs prestations de service. Parmi les méthodes employées figuraient des examens documentaires, des discussions de groupes, des entretiens approfondis; une cartographie des données fondée sur les registres papier; une observation des professionnels de la santé; des ateliers de cocréation avec les soignants; et enfin, des tests d'utilisabilité. Pour terminer, nous avons effectué une analyse de faisabilité observationnelle afin de mesurer le taux d'adhésion à l'application.

**Résultats:**

Les chercheurs ont passé au crible un total de 17 registres papier sur l'ensemble des sites, que nous avons transformés en sept modules repris dans une application numérique compatible avec les appareils mobiles. Ces modules correspondaient aux services proposés, dont le recensement des ménages, les soins prénatals, la planification familiale, la vaccination, la nutrition et la santé infantile. Au total, 65 soignants ont utilisé les modules au cours de l'analyse de faisabilité et le nombre moyen de formulaires soumis chaque semaine était compris entre 8 et 234, en fonction du soignant et de ses responsabilités. Nous avons également observé des variations dans l'utilisation des modules, ce qui montre la nécessité d'assurer un suivi permanent auprès des professionnels de la santé.

**Conclusion:**

Les leçons tirées de cette étude ont permis de façonner des initiatives internationales majeures et d'élaborer un logiciel d'intérêt mondial. Le déploiement de dispositifs numériques requiert des applications bien pensées, une bonne gestion du changement et un renforcement des ressources humaines afin d'obtenir et de préserver les avantages pour le système de santé.

## Resumen

**Objetivo:**

Describir un proceso sistemático que permita transformar los registros en papel en un sistema digital optimizado para mejorar la prestación de servicios y cumplir con los requisitos de información.

**Métodos:**

Se diseñó un estudio formativo en torno a los profesionales de la salud primaria que prestan servicios de salud reproductiva, materna, neonatal e infantil en tres países: de Bangladesh, Indonesia y Pakistán. El estudio se realizódesarrolló entre noviembre de 2014 y junio de 2018. Se desarrolló un prototipo de aplicación digital después de realizar una evaluación sobre las necesidades de las responsabilidades de los profesionales sanitarios, los flujos de trabajo, los requisitos de datos rutinarios y las necesidades de prestación de servicios. Los métodos incluyeron revisiones de documentos, grupos de discusión, entrevistas en profundidad; mapeo de datos de los registros en papel; observaciones de los profesionales sanitarios; talleres de codiseño con los profesionales sanitarios; y pruebas de usabilidad. Por último, se llevó a cabo una evaluación de viabilidad observacional para supervisar la aceptación de la aplicación.

**Resultados:**

Los investigadores revisaron un total de 17 registros en papel en todos los sitios, que se adaptaron a siete módulos dentro de una aplicación digital que funcionaba en dispositivos móviles. Los módulos correspondían a los servicios prestados, como la enumeración de los hogares, la atención prenatal, la planificación familiar, la inmunización, la nutrición y la salud infantil. Un total de 65 profesionales sanitarios utilizaron los módulos durante la evaluación de viabilidad, y la media de envíos de formularios semanales osciló entre 8 y 234, dependiendo del profesional sanitario y de sus responsabilidades. También se observó una variabilidad en el uso de los módulos, lo que requirió un apoyo de seguimiento constante por parte de los profesionales sanitarios.

**Conclusión:**

Las lecciones aprendidas de este estudio dieron forma a iniciativas globales clave y permitieron crear un programa informático de interés mundial. El despliegue de los sistemas digitales requiere aplicaciones bien diseñadas, la gestión del cambio y el fortalecimiento de los recursos humanos para realizar y mantener los beneficios del sistema sanitario.

## ملخص

الغرض وصف عملية منهجية لتحويل السجلات الورقية إلى نظام رقمي مُحسّن لتحسين تقديم الخدمة وتلبية متطلبات إعداد التقارير.

الطريقة قمنا بتصميم دراسة تكوينية عن العاملين في المجال الصحي الأولي، والذين يقدمون خدمات الصحة الإنجابية، وصحة الأم، والمواليد، والأطفال في ثلاث دول هي بنغلاديش وإندونيسيا وباكستان. استمرت الدراسة من نوفمبر/تشرين ثاني 2014 إلى يونيو/حزيران 2018. لقد طورنا تطبيقًا رقميًا أوليًا بعد إجراء تقييم لاحتياجات مسؤوليات العاملين في المجال الصحي، وعمليات سير العمل، ومتطلبات البيانات الروتينية، واحتياجات تقديم الخدمة. وشملت الطريقة المراجعات المكتبية، ومناقشات مجموعات التركيز، والمقابلات المتعمقة؛ وتخطيط بيانات السجلات الورقية؛ وملاحظات العاملين في المجال الصحي؛ والمشاركة في تصميم ورش العمل مع هؤلاء العاملين؛ واختبار قابلية الاستخدام. وأخيرًا، أجرينا تقييمًا للجدوى القائمة على الملاحظة لمراقبة استيعاب التطبيق.

النتائج استعرض الباحثون ما يصل إلى 17 سجلاً ورقيًا عبر المواقع، والتي حولناها إلى سبع وحدات داخل تطبيق رقمي يعمل على الأجهزة المحمولة. تتوافق الوحدات مع الخدمات المقدمة، بما في ذلك التعداد الأسري، والرعاية قبل الولادة، وتنظيم الأسرة، والتحصين، والتغذية، وصحة الطفل. استخدم ما يصل إلى 65 من العاملين في المجال الصحي الوحدات أثناء تقييم الجدوى، وتراوحت عمليات الإدخال الأسبوعية من 8 إلى 234، اعتمادًا على العامل الصحي ومسؤولياته. كما لاحظنا تباينًا في استخدام الوحدات، مما يتطلب دعمًا متسقًا للرصد بواسطة العاملين في المجال الصحي.

الاستنتاج أدى الدرس المستفاد من هذه الدراسة إلى تشكيل المبادرات العالمية الرئيسية، ونتج عنه منفعة عالمية للبرامج. يتطلب نشر الأنظمة الرقمية تطبيقات جيدة التصميم، وإدارة التغيير، وتقوية الموارد البشرية لتحقيق مكاسب النظام الصحي والحفاظ عليها.

## 摘要

**目的:**

旨在描述将纸质登记转变为优化数字系统的系统化过程,以便提高服务水平并满足报告要求。

**方法:**

我们针对在巴基斯坦、孟加拉国和印度尼西亚三个国家提供生殖、孕产妇、新生儿和儿童健康服务的初级卫生保健人员设计了一项形成性研究。该研究在 2014 年 11 月至 2018 年 6 月期间进行。通过对卫生保健人员的职责、工作流程、常规数据要求和服务需求进行需求评估后,我们开发了一个原型数字应用程序。研究方法包括书面材料审查、专题小组讨论、深度采访;数据映射纸质登记内容;观察卫生保健人员;与卫生保健人员共同筹划研讨会;以及可用性测试。最后,我们根据观察结果进行了一项可行性评估,监测应用程序的使用情况。

**结果:**

研究人员审查了所有站点共 17 份纸质登记资料,我们将其转化为在移动设备上运行的数字应用程序中的七个模块。模块与提供的服务相对应,包括家庭统计、产前保健、家庭计划、免疫接种、营养和儿童健康。在可行性评估期间,共有 65 名卫生保健人员使用了这些模块,平均每周提交的表格从 8 个到 234 个不等,具体取决于卫生保健人员及其职责。我们还观察了模块使用的可变性,需要对卫生保健人员进行持续监测。

**结论:**

采用本调研中获得的经验制定关键的全球方案,开发全球性软件产品。部署数字系统时,需要采用精心设计的应用程序、变更管理和加强人力资源建设,才能实现和维持卫生系统的优化管理。

## Резюме

**Цель:**

Описать систематический процесс преобразования бумажных реестров в цифровую систему, оптимизированную с целью улучшения предоставления услуг и выполнения требований к отчетности.

**Методы:**

Авторы разработали развивающее исследование, в котором приняли участие работники первичной медико-санитарной помощи, предоставляющие услуги в сфере репродуктивного здоровья, здоровья матерей, новорожденных и детей в трех странах: Бангладеш, Индонезии и Пакистане. Исследование проводилось с ноября 2014 года по июнь 2018 года. После проведения оценки потребностей медицинских работников, рабочих процессов, рутинных требований к данным и потребностей в предоставлении услуг авторы разработали прототип цифрового приложения. Методы включали анализ документации, дискуссии в фокус-группах, углубленные собеседования, привязку данных бумажных реестров, наблюдения за медицинскими работниками, разработку семинаров совместно с работниками здравоохранения и тестирование практичности. Наконец, авторы провели наблюдательную оценку осуществимости, чтобы отслеживать уровень применения приложения.

**Результаты:**

Исследователи просмотрели в общей сложности 17 бумажных реестров во всех центрах, которые были преобразованы в семь модулей в цифровом приложении, работающем на мобильных устройствах. Модули соответствовали предоставляемым услугам, включая регистрацию домохозяйств, дородовую помощь, планирование семьи, иммунизацию, питание и здоровье детей. В общей сложности 65 медицинских работников использовали модули во время оценки осуществимости, и в среднем еженедельно заполнялось от 8 до 234 форм в зависимости от медицинского работника и его обязанностей. Авторы также пронаблюдали разницу в использовании модулей, что означает необходимость постоянной мониторинговой поддержки медицинских работников.

**Вывод:**

Опыт, полученный из этого исследования, сформировал основные глобальные инициативы и привел к глобальному благу программного обеспечения. Внедрение цифровых систем требует хорошо разработанных приложений, управления изменениями и укрепления кадровых ресурсов для реализации и сохранения преимуществ системы здравоохранения.

## Introduction

Global agencies advocate for the use of information and communication technologies to accelerate progress on priorities, such as the sustainable development goals, the Roadmap for Measurement and Accountability, and the Global Strategy for Women’s, Children’s and Adolescents’ Health.^1^^,^^2^ In particular, the transition from paper to digital systems for routine health information systems and managing service delivery has attracted major investment.^3^^–^^12^ Some of the drivers for this transition include the clerical burden of maintaining multiple paper-based registers and the manual aggregation of data that absorbs time that could otherwise be used for service provision and supervision. Developing reports from paper-based data is not only error-prone and time-consuming but also risks propagation of these problems across several administrative levels and limiting opportunities for real-time, data-driven decision-making.^8^^,^^10^^–^^13^ Lastly, the current design of paper-based systems places a greater emphasis on data collection for health management information systems and indicator reporting,^14^ rather than on point-of-care decision-support needs, which are important for high-quality care.

Although there is heightened interest in the digital transformation of paper-based systems, this process may be conducted in an ad hoc manner and protracted due to the scale of the challenge. Often the focus may be to convert analogue forms into electronic versions, as opposed to digitalizing, which introduces opportunities for improvements and harnesses the potential added value from digital systems, such as real-time decision support.^15^^,^^16^ At the individual level of service delivery, health workers may value the familiarity and convenience of paper registers^14^ and be apprehensive of the changes associated with – and consequences of – shifting to digital tools. Health workers’ varying levels of literacy in information and communication technologies may contribute to potential resistance to transitioning to digital tools. Additionally, digital systems that are not responsive to users’ needs may hamper the extent to which paper-based tools can be migrated successfully into a digital format.^17^^–^^23^ Despite the abundance of research on barriers to and facilitators for the deployment of digital systems,^17^^–^^23^ the literature is limited in detailing the processes of how to digitalize.

In 2014 the World Health Organization (WHO) launched a multisite study across Bangladesh, Indonesia and Pakistan to validate a replicable and systematic process of transforming paper registers to digital systems that are optimized to enhance service provision and fulfil reporting requirements. Here we describe the methods used to create the digital system and a preliminary assessment of its feasibility and uptake. We highlight key lessons from this effort and offer reflections towards a pragmatic approach for the digital transformation of paper registers.

## Methods

### Study design

We designed a multisite, formative study with a common set of research questions across sites. Table 1 provides an overview of the three phases of developing and piloting the digital register: (i) needs assessment; (ii) prototype development; and (iii) feasibility assessment.^24^ The study ran from November 2014 to June 2018. The study was conducted by the Technologies for Health Registers, Information, and Vital Events group comprised of researchers, technology developers and government partners. Each country team was interdisciplinary, comprising a local technology team embedded within the research teams to co-design the implementations. Ona, a software development company in Nairobi, Kenya, provided the technology development guidance and assisted site teams to develop and deploy their own software. WHO provided overall coordination, including protocol development, standardizing templates for extracting and analysing information and facilitating information exchange and learning across sites.

**Table 1:** Research phases and questions in the study of transitioning paper registers to digital systems, Bangladesh, Indonesia and Pakistan, 2015–2018

Phase	Aims	Research questions	Methods
Phase 1: Needs assessment	Understand health workers' responsibilities, workflows, routine data reporting needs, and intervention schedules and tasks for service delivery to establish the functional requirements for an optimized digital register system	What are the current workflows, data generation responsibilities and technology capacities of health workers in each study site? What are the current information systems and data needs of supervisors and decision-makers in each study site? How can existing workflows, data collection and data reporting be optimized through a digital application?	Desk review; data mapping; observation of staff; in-depth interviews and focus group discussions; semi-structured questionnaires
Phase 2: Prototype development	Create a functional version (minimum viable product) of digital application that meets minimum requirements of capturing routinely collected data, addresses service delivery needs, and achieves usability across targeted cadres of health worker	What functions are required of the digital register for both the generic version and the local adaptations for each site and cadre of health worker? What are the key factors to make the digital register easy to use and to integrate the workflows, data collection and reporting needs of key cadres of health workers, supervisors and decision-makers? What are the technical requirements to support local adaptation and use of the digital register?	Focus group discussions; usability testing; co-design workshops; repeated feedback to refine prototype
Phase 3: Feasibility assessment	Determine the factors necessary to support implementation of the digital system and uptake by health workers	What is the uptake by health workers of the developed digital register? What are the requirements to support implementation of the digital register?	Pilot testing with continuous data monitoring; monitoring of project data

### Study setting

The study was conducted among primary health workers providing reproductive, maternal, newborn and child health services, and their supervisors and managerial health staff, in three country sites: Gaibandha district (rural), Bangladesh; Central Lombok district (rural), Indonesia; and Karachi (urban) and Shikarpur (rural) districts, Pakistan. All sites were in countries that prioritized digitalizing the routine health information systems and which had a strong reliance on primary health workers to provide the targeted health services. However, each site had different information needs, models of service delivery, cadres of health workers and health infrastructure capabilities, to provide a diverse range of experiences to inform the digitalization process. Research teams engaged their respective district health office and health ministry counterparts to ensure that the process and findings supported a broader digital health vision and informed plans for sustainability beyond the study. 

### Intervention

The first phase (running for 6‒9 months, depending on the site) was a needs assessment to gather information on health workers’ responsibilities, workflows, routine data reporting requirements and service delivery needs. Researchers at each site conducted a desk review of all the paper registers for all relevant cadres of health workers listed in Table 2. We then consolidated and cross-checked the findings for logical ways of grouping together the multiple registers. We coded the data fields from the paper registers using data extraction forms^25^ to describe the purpose of the data elements and determine the data points that would need to be incorporated into the digital system. The extraction forms detailed the frequency of the data collected; type of data field (numeric, string or multiple choice); purpose of the information (service delivery or aggregation); how the data were aggregated and linked to indicators; and abnormalities or issues with collecting the data (such as data fields that were repeated or redundant, meaning they did not contribute to service delivery or reporting needs). Based on this extraction, research teams at sites compiled a spreadsheet data dictionary following a standardized template.

**Table 2:** Study participants by cadre type and setting in three study sites in Bangladesh, Indonesia and Pakistan, 2015–2018

Participants	Health worker cadres by setting		
	Gaibandha district, Bangladesh	Central Lombok district, Indonesia	Karachi (urban) and Shikarpur (rural) districts, Pakistan
Primary health workers (5–7 of each cadre)	**Family welfare assistants**: provide routine reproductive, maternal, newborn and child health services through home visits, such as antenatal screening and referrals for clinical services **Family welfare visitors**: provide routine reproductive, maternal, newborn and child health services through clinics, with a focus on family planning and antenatal care **Health assistants**: deliver immunizations according to the expanded programme on immunizations; hold routine outreach vaccination events	**Midwives** *(bidan):* provide antenatal care; conduct deliveries, intrapartum care and postnatal care; provide contraception, including intrauterine devices **Nutritionist workers** *(gizi):* provide services related to maternal and child nutrition promotion; child growth monitoring; distribution of vitamin A; community outreach for anthelmintics and nutrition **Vaccinators** *(vaksinator):* provide routine child vaccinations at health outreach centres and school programmes	**Lady health workers**: provide core community activities, such as household registration and census enumeration; pregnancy surveillance; community-based antenatal care; polio vaccination and other routine immunizations; vital events tracking; and related reproductive, maternal, newborn and child health services **Vaccinators**: provide all childhood vaccinations and women’s tetanus vaccinations at clinics and during outreach services; responsible for tracking logistics and supplies
Supervisors of primary health workers (3–5 of each cadre)	**Family planning inspectors**: supervise family welfare assistants **Health inspectors**: supervise health assistants	**Head of primary health care**: supervise and be responsible for all health workers at the primary health-care centre (*puskesmas*) ***bidan* coordinator***:* supervise *bidan* midwives ***gizi* coordinator***:* supervise nutrition workers ***vaksinator* coordinator***:* supervise vaccinators	**Lady health visitors**: supervise lady health workers; provide facility-based antenatal care, deliveries; handle obstetric complications
District-level health managers for reproductive, maternal, newborn and child health services (1 of each cadre)	Deputy director family planning; *Upazila* health and family planning officer; *Upazila* family planning officer	District health office head; District public health head; District family health division head; District midwife (maternal and child health) coordinator; District vaccinator coordinator; District nutritionist coordinator	Provincial head of expanded programmes on immunization; Provincial director of lady health worker programme

Notes: *Upazila* are the equivalent of subdistricts. Local terms for the health worker cadres are shown in italics.

We also conducted in-depth interviews with the relevant cadres of health worker and their respective supervisors to further determine their requirements related to routine data collection, service provision needs, intervention schedules, and circumstances that call for deviations from their standard workflows. Site teams also shadowed health workers to observe the workers’ interactions and work patterns in real time. During direct observations, a trained study assistant made notes of tasks performed, when and how data were recorded, and how data were used by health workers to make decisions during interactions with clients. 

The second phase, over the following 6‒9 months, was to develop a prototype digital application that met the identified needs for health service delivery and information systems.^26^ Site teams used paper mock-ups in co-design workshops as a way for end-users to provide input on and collaboratively refine the digital prototype. Our intention was to identify new features at an early stage (client scheduling, sorting entries by name, alerts for conditions requiring following) as well as issues of functionality and usability.^27^ The teams also embedded analytical tools, such as Flurry Analytics (Flurry, San Francisco, United States of America) to track commonly used functions and steps that led to system crashes or errors in the software application, as part of measures for improving the usability of the digital application.

We used the Open Smart Register Platform (OpenSRP, Nairobi, Kenya), an open-source, mobile-based digital application for developing the prototypes, testing the usability and conducting the feasibility assessment. Each site customized OpenSRP and organized the content as modules corresponding to the health domain and the service delivery needs of the health worker cadre. Once a functional prototype was developed, the site teams assessed health workers’ ease of completing tasks on the digital application by observing and timing how health workers reacted to the various functions assigned to them, such as registering a client and completing a consultation. We also used focus group discussions to obtain additional information on health workers’ preferences and challenges with the prototypes and to understand their requirements for additional features. 

### Implementation

The third phase was the feasibility assessment. Each site implemented a version of the digital application developed based on their site-specific requirements and organized across health domain modules and responsibilities of the targeted health worker cadre. We monitored uptake of the developed digital application over a 9-month period at the Indonesia site (June 2015 to March 2016) and over 5 months at the Bangladesh and Pakistan sites (November 2016 to March 2017). We measured uptake based on successful enrolment of new clients or households done through the application, as well as weekly health worker form submissions. During the feasibility assessment, sites continued to update the digital application modules in response to feedback from the health workers.

Each site team conducted a 5-day training for targeted cadres of health worker (Table 2) on the use of the mobile device (such as turning on or off the data plan), the functions of the digital application (such as following up clients, interpreting prompts) and in some cases refresher training on the health content (such as vaccination schedules). The training also included use of dummy data and scenarios to test the practical uses of the application.

Site teams developed a system monitoring plan, in which technical supervisors tracked the mobile devices using centralized system monitoring software and a review of form submissions and time stamps. All health workers received a unique username and password login detail for accessing the application modules on a tablet device. Site teams tracked failures of the mobile devices, evidence of tampering and staff members’ active times at the individual user level and compared the data across the pool of users. Users who deviated markedly from the group median and mean number of forms submitted were visited by supervisory field teams to verify the information. We established specific data collection benchmarks based on catchment populations to determine the weekly proportion of clients met in a timely manner and of appointments overdue and appointments expired (no longer valid) to identify high and low-performing workers. Site teams reviewed a random selection of forms submitted by health workers for technical validity (such as plausibility of correct last menstrual period) and calculated potential error rates. The teams also generated statistical summary sheets for supervisory review and identification of performance variability. 

The multisite study team met in person annually to review progress and held bi-weekly online meetings to exchange lessons learnt and discuss solutions to common challenges. Two cross-site workstreams emerged. One workstream focused on the technology and overall design of the digital system, including how to adapt the application to allow integration with other digital systems and manage the data types and coding language of the application. Another workstream focused on questions related to the content, training, deployment and monitoring. At the end of the study, WHO staff consolidated the health content across different sites to work towards a generic application for each health domain.

We extracted data via secure data transmission protocols to external dashboards and reporting tools for monitoring and evaluation purposes. Individual and patient data captured by health workers during routine service delivery were made available to the relevant government health management information reporting systems, in line with the standard practice of paper reporting forms.

Ethical approval for the study was obtained from the WHO ethical review committee and the institutional review boards of Interactive Research and Development, Pakistan, Bangladesh Medical Research Council, Johns Hopkins School of Public Health, USA and University of Mataram, Indonesia. 

The Review Panel on Research Projects, coordinated by the WHO Department of Sexual and Reproductive Health and Research and the Special Program on Human Reproduction approved the scientific and technical content of the core research protocol through an external committee (Protocol ID A65886).

## Results

Study teams reviewed an average of 17 paper registers at each site, which we consolidated into seven modules within the digital application for different cadres of health worker across different sites. Two modules were designed for the Bangladesh site (for family welfare assistants and family welfare visitors); three modules for the Indonesia site (for midwives, nutritionists and vaccinators); and two modules for the Pakistan site (for lady health workers and vaccinators; Table 3). We made efforts to streamline the numerous registers into health domain modules on, for example, antenatal care, household enumeration and vaccination. The common core functions of the digital application included: (i) tracking of client health status and services received; (ii) decision support to health workers by providing prompts and alerts; (iii) screening for potential risks; (iv) identifying clients in need of services; (v) scheduling follow-up appointments in accordance with the clinical protocols; and (vi) coordinating care across health workers. The development process resulted in features for enhancing usability, such as identifying returning clients through quick response (QR) codes, performing a smart search to facilitate follow-up of clients, printing out completed forms to submit for reporting, enabling health workers to view their overall performance, and using the application offline. Screenshots of these modules and outputs are provided in the supplementary files in the authors’ data repository.^25^

**Table 3:** Description of the paper registers reviewed and digital modules developed at three study sites in Bangladesh, Indonesia and Pakistan, 2015–2018

Stage	Gaibandha district, Bangladesh	Central Lombok district, Indonesia	Karachi and Shikarpur districts, Pakistan
**Stage 1: Needs assessment**			
Register review	22 paper registers reviewed	20 paper registers reviewed:	16 paper registers reviewed:
	Family welfare assistants’ registers: couple roster; child roster (age 0–1 years); child roster (age 0–5 years); adolescent health service delivery roster; pregnant woman roster; household population roster; 2-monthly summary reporting forms for family welfare assistants Family welfare visitors’ registers: antenatal care register; delivery register; child register; general patient register; 3 summary reporting forms Health assistants’ registers: adolescent girl and woman registration and vaccination information; neonatal registration and vaccination information; daily vaccine and other care reports	Antenatal care registers: new antenatal care; antenatal care visit; birth plan; delivery and postnatal care Family planning registers: new family planning method form; updated family planning method form; closed family planning method form Postnatal care registers: postnatal care registration; postnatal care visit; postpartum family planning Child registry registers: child registration; neonatal visit; baby visit; child visit; child immunization	Lady health workers’ registers: family register; monthly report by lady health workers; community chart; register for diagnosis; family planning register; lady health workers monthly programme register; health committee meeting report; women’s group meeting report; list of children less than 3 years of age; list of pregnant women Lady health visitors’ registers: child health register; outpatient department register; stock register Vaccinators’ registers: daily register; permanent register; stock register
No. of professionals interviewed or observed^a^	15 health workers observed: 5 family welfare assistants 5 family welfare visitors 5 health assistants	23 health workers interviewed or observed: 5 community-based midwives (*bidan*) 5 nutritionists (*gizi*) 5 vaccinators (*jurim*) 6 community health volunteers (*kader*) 1 medical chief or head of Central Lombok district health office 1 district midwife coordinator	19 health workers interviewed or observed: 5 vaccinators 5 lady health workers 5 lady health visitors 1 lady health supervisor 1 deputy provincial head of the expanded programme on immunization 1 provincial director of the lady health workers programme 1 deputy provincial director of lady health visitor programme
**Stage 2: Prototype development**			
No. of modules	2 digital prototypes	3 digital prototypes	2 digital prototypes
Details	A module for family welfare assistants to record community health worker activities related to household registration, pregnancy registration, antenatal care danger signs, referral and commodity distribution, and postnatal care; a module for family welfare visitors to record antenatal care	A module for community-based midwives to record registration of mothers, antenatal care, postnatal care, care for children under 5 years of age, and family planning; a module for nutritionists to record child registration, anthropometry and anaemia tracking, electronic growth charts, nutrition education and promotion media; a module for vaccinators that included child registration, child expanded programme on immunization schedule and vaccine tracking	A module for lady health workers to register households including women and children for pregnancy registration, antenatal and postnatal care and family planning; a vaccinator module for registration and follow-up of women and children at expanded programme on immunization centres and immunization clinics
**Stage 3: Feasibility assessment**			
No of health workers trained on the module	45 family welfare assistants; numbers were increased from the research protocol on request from the health ministry 18 supervisors of family welfare assistants	13 midwives 12 nutritionists 12 vaccinators	6 vaccinators 3 lady health workers
No. of health workers using the module during pilot deployment	44 family welfare assistants	13 midwives 12 nutritionists 12 vaccinators	8 health workers (3 lady health workers and 5 vaccinators)

^a^ Only health workers involved in service delivery were observed; supervisory staff were only interviewed.

Note: Local terms for the health worker cadres are shown in italics.

The process of mapping registers across the range of health domains demonstrated the repetition of the data collected by different cadres of health workers. For example, in Bangladesh, of the 571 data fields appearing across different registers for the family welfare assistants, we estimated that less than 100 data points were necessary for the health records, after removing duplicate and redundant fields.^28^ Likewise, in Indonesia we found that 33 of 87 (38%) indicators compiled from data collected by midwives, vaccinators, nutritionists and community child development workers were repeated or redundant, or were not compatible. We streamlined redundant and conflicting data elements to identify the essential requirements for the digital prototype and conducted a series of stakeholder discussions to gain consensus on the final set of data elements to be included.^28^

We trained a total of 67 health workers across the three sites on the use of the application and monitored its uptake by 65 health workers during the feasibility assessment (Table 3). Table 4 shows the numbers of households and patients registered into the application over the trial period and the usage statistics for the health workers. 

**Table 4:** Outcomes of the feasibility assessment during pilot deployment of a new digital records application in three study sites in Bangladesh, Indonesia and Pakistan, 2016–2017

Indicator	Gaibandha district, Bangladesh (Nov 2016–Mar 2017)	Central Lombok district, Indonesia (Jun 2015–Mar 2016)	Karachi and Shikarpur districts, Pakistan (Nov 2016–Mar 2017)
**All sites**			
No. of registrations into the module	1348 households registered by family welfare assistants; 1402 pregnant women enrolled by family welfare assistants	959 new pregnant women registered by midwives (mean per midwife: 73; range: 10–346); 180 new family planning users enrolled (mean per midwife: 13; range: 0–159); 137 new children registered (mean per midwife: 10; range: 0–56)	334 households (with 1762 household members) enrolled by lady health workers; 438 children from these households referred and vaccinated
**Site-specific performance monitoring**			
Mean number of forms submitted per health worker per week (range)	48 forms (8–101)	54 forms (26–234)	NA
No. (%) of health workers submitting forms consistently for at least 10 days	27/44 (61)	8/13 (62)	NA
No. (%) of health workers with form submissions below the average	17/44 (39)	5/13 (38)	NA
Mean number of sessions on use of modules per health worker (range)	NA	193 (67–501)	NA
Time taken for household enrolment, minutes (range)	NA	NA	Mean: 2.5; median: 1.8 (0.8–13.3)

NA: not applicable.

**Fig. 1:**
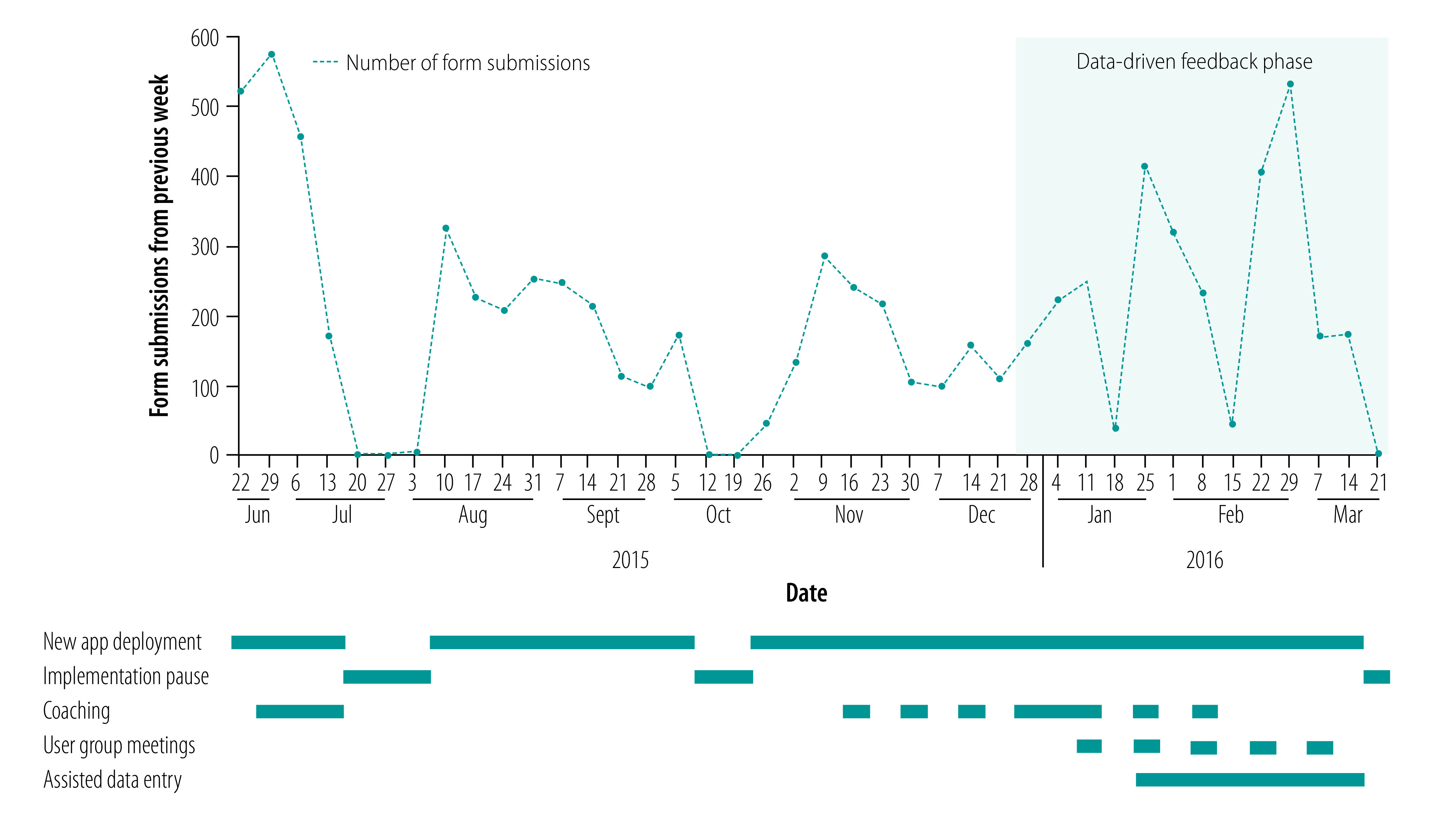
Changes in digital form submissions by nurse midwives in response to feedback and coaching, Central Lombok district, Indonesia, June 2015 to March 2016

The majority of health workers were able to use the digital modules but with substantial variability in the numbers of form submissions. The weekly number of forms submitted by health workers ranged from 8 to 234, and depended on the responsibilities of the cadre of health worker, including whether they were community- or facility-based (Table 4). This variation persisted throughout the feasibility assessment and highlighted the need for data-driven feedback and coaching to optimize uptake of the digital system by health workers. For example, when reinforcement and supervision was provided at the Indonesia site, the rates of form submissions improved (Fig. 1). Additional examples from the Bangladesh and Pakistan sites are available in the data repository.^25^

App: application

Notes: The large declines in forms submitted were typically due to periodic trainings and health centre events that workers needed to attend, and therefore made fewer form submissions. The pauses correspond to times when the module was not in use, such as public holidays.

## Discussion

Although the process was resource intensive, we were able to create digital application modules that were responsive to a diverse set of health worker cadres and continue to be implemented in different countries beyond the research period. The study also provided important information for WHO and research teams beyond the observed findings. The approach used in Indonesia directly informed the transformation of the WHO antenatal care guidelines into the WHO digital antenatal care module, a digital decision-support and client-record application.^29^ Most notably, this study catalysed the launch of the WHO SMART guidelines initiative^30^ to provide generic recommended health content in a format that can be used within digital systems.^30^ Lastly, the process of refining OpenSRP contributed to its recognition as a digital global good,^31^ and OpenSRP continues to evolve and be implemented globally.

Throughout the study, several common themes emerged around the sustainability of this effort. First, the digitalization process required careful planning to link with the local health information systems and existing digital systems, and putting measures in place to adapt to the evolving health information system. For example, in Bangladesh, teams spent approximately 9 months in the initial phase, mapping the different digital tools used and working with government counterparts to ensure there was a plan for information exchange across systems. With the introduction of data standards, the process for digital systems to exchange data may become more efficient. In Indonesia, the team drew on lessons learnt from the study to introduce the Fast Healthcare Interoperability Resources data standard^32^ to the health ministry as a way to exchange data more efficiently in digital systems.

The concurrent use of paper and digital systems was another recurring issue as countries continued to require paper-based reporting. An interim strategy developed in consultation with government partners in Indonesia was to use data entry assistants to complete the paper registers, while the health workers focused on using the digital modules. Additionally, the Indonesian site undertook a process of entering information from existing registers so that health workers could have immediate access to the details for follow-up care of pregnant women. In Pakistan, financial incentives were used with success but issues were raised about the long-term viability of this approach. Although we were not able to resolve the balancing of the dual use of paper and digital systems, we identified approaches to alleviate the double burden. Further work is needed to assist governments in managing this mix of paper and digital systems, as well as understanding the requirements, cost implications and level of uptake by health workers if digital systems become the primary source. 

The adoption of the digital application also required extensive training and time to allow health workers to familiarize themselves with the modules, particularly for older health workers. The close monitoring of uptake of the application assisted in targeting feedback, and sometimes uncovered underlying issues, such as death in a health worker’s family, thus explaining the individual’s divergent performance. In Pakistan, individual attention in the training was useful for enhancing health workers’ understanding of the application. Although most of the health workers could use the application with limited supervision by the end of the training, a follow-up plan was made for those still facing difficulties. In Bangladesh, the research team dedicated a 90-day period for health workers to familiarize themselves with the digital application and for the research team to solve minor problems and enhance the usability of the application. The team also developed a checklist to systematically review health workers’ use of the digital application before the system was deployed in real situations. In Indonesia, the research team monitored health workers’ daily patterns of use, including challenges faced during the deployment. Coaching on their performance in service coverage as well as active feedback from the health workers on how to improve the platform allowed for rapid modifications to the digital application, with the goal of improving uptake of the modules. Additionally, considerable efforts were dedicated to the use of digitized data to facilitate monthly reporting and active use of data by supervisors in monthly review meetings.

When this study was initiated, the use of mobile technologies for expanding health information systems was met with enthusiasm by implementers and funders, despite the limited evidence base for feasibility or impact.^33^^–^^37^ Furthermore, with the exception of the Collaborative Requirements Development Methodology^38^ developed in the United States, there were few resources to guide the transformation of paper registers into digital systems. Since then, investments in digitalization and national-level implementations have greatly increased. Nevertheless, lessons learnt from this process remain relevant: the political and technical considerations for streamlining data capture, managing the dual burden of using both paper and digital systems, and facilitating appropriate and continuous support to health workers.

Our experiences also resonate with commonly documented challenges in digitalization initiatives, such as aligning with the end-user needs and workflows, ensuring training and reinforcement to health workers, and planning for successful information exchange and data use.^4^^,^^10^^,^^39^^–^^44^ We also encountered issues that emerged more clearly during the digitalization process. An example was the management of incomplete information (date of birth or unnamed children), which existed on paper registers but needed to be addressed in the digital system due to the built-in checks and compulsory fields. The fragmentation of service delivery processes within health systems was also an impediment to designing a digital system appropriate to clients’ needs, but also presented an opportunity to identify ways in which digital tools can help synchronize and coordinate care across disparate primary health workers. Ultimately, the deployment of digital systems requires both well-designed applications and human resource development plans to realize and sustain the gains to the health system; digital modules are necessary, but not sufficient, for successful digitalization.

Although this was a multisite study, one limitation is that all the sites were in south-eastern Asia and did not include settings from other geographical areas. Furthermore, the data span across different time periods due to contextual factors (such as local ethical approval, the process to gain access to health workers and alignment with government priorities). Lastly, while this study aimed to offer a systematic approach to digitalization, there were inevitable adaptations and local deviations from the generic protocol required to accommodate government priorities and engagements across different stakeholders.

In conclusion, the digitalization of routine health information systems requires a systematic approach and concerted efforts with governments to move beyond simply automating the existing system and to manage the accompanying organizational, behavioural and technical transitions. As digital technologies become increasingly widespread, the mechanics of effectively designing, implementing and sustaining digital systems for service provision and accountability remains an area for continuous learning.

## References

[1] Roadmap for measurement and accountability. Washington, DC: World Bank, World Health Organization, United States Agency for International Development; 2015. Available from: https://www.healthdatacollaborative.org/fileadmin/uploads/hdc/Documents/the-roadmap-for-health-measurement-and-accountability.pdf [cited 2021 Dec 28].

[2] The global strategy for women’s, children’s and adolescents’ health (2016‒2030). New York: United Nations; 2015. Available from: https://globalstrategy.everywomaneverychild.org/ [cited 2021 Dec 28].

[3] Adamou B, Barden-O’Fallon J, Williams K, Selim A. Routine family planning data in the low- and middle-income country context: a synthesis of findings from 17 small research grants. Glob Health Sci Pract. 2020 Dec 23;8(4):799–812. 10.9745/GHSP-D-20-00122 33361243 PMC7784078

[4] Recommendations on digital interventions for health system strengthening. Geneva: World Health Organization; 2020. Available from: https://www.who.int/publications/i/item/9789241550505 [cited 2021 Dec 28]. 31162915

[5] National digital health blueprint. New Delhi: Government of India, Ministry of Health Family Welfare; 2020. Available from: https://main.mohfw.gov.in/sites/default/files/Final%20NDHB%20report_0.pdf [cited 2021 Dec 20].

[6] Uganda national eHealth strategy 2017–2021. Kampala: Republic of Uganda Ministry of Health; 2017. Available from: https://health.go.ug/sites/default/files/National%20e_Health%20Strategy_0.pdf [cited 2022 Dec 20].

[7] WHA71. 7 Digital health. Resolutions and decisions annexes. In: Seventy-first World Health Assembly, Geneva, 21–26 May 2018. Geneva: World Health Organization; 2018. Available from: https://apps.who.int/gb/ebwha/pdf_files/WHA71-REC1/A71_2018_REC1-en.pdf#page=1 [cited 2022 Dec 20].

[8] Gera R, Muthusamy N, Bahulekar A, Sharma A, Singh P, Sekhar A, et al. An in-depth assessment of India’s mother and child tracking system (MCTS) in Rajasthan and Uttar Pradesh. BMC Health Serv Res. 2015 Aug 11;15(1):315. 10.1186/s12913-015-0920-2 26259836 PMC4530478

[9] Tanzania digital health investment road map 2017‒2023. Dodoma: United Republic of Tanzania Ministry of Health and Social Welfare; 2017. Available from: https://www.path.org/resources/tanzania-digital-health-investment-road-map-2017-2023/ [cited 11 Jul 2022].

[10] Kodkany BS, Derman RJ, Honnungar NV, Tyagi NK, Goudar SS, Mastiholi SC, et al. Establishment of a maternal newborn health registry in the Belgaum district of Karnataka, India. Reprod Health. 2015;12(S2) Suppl 2:S3. 10.1186/1742-4755-12-S2-S3 26062791 PMC4464217

[11] Frøen JF, Myhre SL, Frost MJ, Chou D, Mehl G, Say L, et al. eRegistries: electronic registries for maternal and child health. BMC Pregnancy Childbirth. 2016;16(1):279. 10.1186/s12884-016-0801-7 26791790 PMC4721069

[12] Nguyen NT, Vu HM, Dao SD, Tran HT, Nguyen TXC. Digital immunization registry: evidence for the impact of mHealth on enhancing the immunization system and improving immunization coverage for children under one year old in Vietnam. mHealth. 2017 Jul 19;3:26. 10.21037/mhealth.2017.06.03 28828373 PMC5547172

[13] George A. ‘By papers and pens, you can only do so much’: views about accountability and human resource management from Indian government health administrators and workers. Int J Health Plann Manage. 2009 Jul-Sep;24(3):205–24. 10.1002/hpm.986 19384895

[14] Bosch-Capblanch X, Oyo-Ita A, Muloliwa AM, Yapi RB, Auer C, Samba M, et al. Does an innovative paper-based health information system (PHISICC) improve data quality and use in primary healthcare? Protocol of a multicountry, cluster randomised controlled trial in sub-Saharan African rural settings. BMJ Open. 2021 Jul 29;11(7):e051823. 10.1136/bmjopen-2021-051823 34326056 PMC8323359

[15] Gartner glossary: digitalization [internet]. Stamford: Gartner Inc.; 2022. Available from: https://www.gartner.com/en/information-technology/glossary/digitalization [cited 2021 May 5].

[16] Tamrat T, Ratanaprayul N, Barreix M, Tuncalp O, Lowrance D, Thompson J. Transitioning from paper to digital systems: the role of WHO digital adaptation kits (DAKs) in operationalizing recommendations and standards. Glob Health Sci Pract. 2022 Feb 28;10(1):e2100320. 10.9745/GHSP-D-21-00320 35294382 PMC8885357

[17] Dolan SB, Alao ME, Mwansa FD, Lymo DC, Bulula N, Carnahan E, et al. Perceptions of factors influencing the introduction and adoption of electronic immunization registries in Tanzania and Zambia: a mixed methods study. Implement Sci Commun. 2020 Mar 30;1(1):38. 10.1186/s43058-020-00022-8 32885195 PMC7427960

[18] Duong H, Dao S, Dang H, Nguyen L, Ngo T, Nguyen T, et al. The transition to an entirely digital immunization registry in Ha Noi Province and Son La Province, Vietnam: readiness assessment study. JMIR Form Res. 2021 Oct 25;5(10):e28096. 10.2196/28096 34694232 PMC8576599

[19] Jacob N, Rice B, Kalk E, Heekes A, Morgan J, Brinkmann S, et al. Consolidating strategic information to monitor progress against the UNAIDS 90-90-90 targets: evaluating the operational feasibility of an electronic HIV testing register in Cape Town, South Africa. BMC Health Serv Res. 2020 Aug 6;20(1):720. 10.1186/s12913-020-05517-7 32762660 PMC7409395

[20] Kaphle S, Chaturvedi S, Chaudhuri I, Krishnan R, Lesh N. Adoption and usage of mhealth technology on quality and experience of care provided by frontline workers: observations from rural India. JMIR Mhealth Uhealth. 2015 May 28;3(2):e61. 10.2196/mhealth.4047 26023001 PMC4464193

[21] Mvundura M, Di Giorgio L, Lymo D, Mwansa FD, Ngwegwe B, Werner L. The costs of developing, deploying and maintaining electronic immunisation registries in Tanzania and Zambia. BMJ Glob Health. 2019 Nov 25;4(6):e001904. 10.1136/bmjgh-2019-001904 31803511 PMC6882552

[22] Ovretveit J, Nelson E, James B. Building a learning health system using clinical registers: a non-technical introduction. J Health Organ Manag. 2016 Oct 10;30(7):1105–18. 10.1108/JHOM-06-2016-0110 27700477

[23] Fritz F, Tilahun B, Dugas M. Success criteria for electronic medical record implementations in low-resource settings: a systematic review. J Am Med Inform Assoc. 2015 Mar;22(2):479–88. 10.1093/jamia/ocu038 25769683 PMC11737103

[24] Principles for digital development [internet]. Washington, DC: Digital Impact Alliance; 2022. Available from: https://digitalprinciples.org/ [cited 2020 Nov 18].

[25] Tamrat T, Chandir S, Alland K, Pedrana A, Shah MT, Footitt C, et al. Digitalization of routine health information systems; Bangladesh, Indonesia, Pakistan. London: figshare; 2022. 10.6084/m9.figshare.20462046.v1 10.6084/m9.figshare.20462046.v1 PMC9511663 36188022

[26] Martin B. Universal methods of design: 100 ways to research complex problems, develop innovative ideas, and design effective solutions. Beverly: Rockport Publishers; 2012.

[27] Lidwell W, Holden K, Butler J, Elam K. Universal principles of design: 125 ways to enhance usability, influence perception, increase appeal, make better design decisions, and teach through design. Beverly: Rockport Publishers; 2010.

[28] Masters J, Labrique A. Improving service delivery in rural Bangladesh through CHW process and data flow analysis. Baltimore: Johns Hopkins University School of Public Health; 2014.

[29] Haddad SM, Souza RT, Cecatti JG, Barreix M, Tamrat T, Footitt C, et al. Correction: building a digital tool for the adoption of the World Health Organization’s antenatal care recommendations: methodological intersection of evidence, clinical logic, and digital technology. J Med Internet Res. 2020 Oct 13;22(10):e24891. 10.2196/24891 33048822 PMC7592070

[30] Mehl G, Tunçalp Ö, Ratanaprayul N, Tamrat T, Barreix M, Lowrance D, et al. WHO SMART guidelines: optimising country-level use of guideline recommendations in the digital age. Lancet Digit Health. 2021 Apr;3(4):e213–6. 10.1016/S2589-7500(21)00038-8 33610488

[31] Digital Square investments in global goods: approved global goods [internet]. Washington, DC: Digital Square; 2022. Available from: https://digitalsquare.org/resourcesrepository/global-goods-guidebook [cited 2022 May 22].

[32] Health Level 7 Fast Health Interoperability Resources [internet]. Ann Arbor: HL7 FHIR Foundation; 2022. Available from: https://fhir.org/ [cited 2022 Jul 18].

[33] Agarwal S, Perry HB, Long LA, Labrique AB. Evidence on feasibility and effective use of mHealth strategies by frontline health workers in developing countries: systematic review. Trop Med Int Health. 2015 Aug;20(8):1003–14. 10.1111/tmi.12525 25881735 PMC4692099

[34] Aranda-Jan CB, Mohutsiwa-Dibe N, Loukanova S. Systematic review on what works, what does not work and why of implementation of mobile health (mHealth) projects in Africa. BMC Public Health. 2014 Feb 21;14(1):188. 10.1186/1471-2458-14-188 24555733 PMC3942265

[35] Labrique A, Vasudevan L, Chang LW, Mehl G. H_pe for mHealth: more “y” or “o” on the horizon? Int J Med Inform. 2013 May;82(5):467–9. 10.1016/j.ijmedinf.2012.11.016 23279850 PMC3849805

[36] Planning an information systems project: a toolkit for public health managers. Seattle: PATH; 2013. Available from: https://media.path.org/documents/TS_opt_ict_toolkit.pdf [cited 2021 Dec 2021].

[37] Gartner hype cycle: interpreting technology hype [internet]. Stamford: Gartner Inc; 2020. Available from: https://www.gartner.com/en/research/methodologies/gartner-hype-cycle [cited 2021 Jul 6].

[38] Collaborative requirements development methodology (CRDM): a collaborative approach to public health systems [internet]. Decatur: Public Health Informatics Institute; 2020. Available from: https://www.phii.org/crdm [cited 2020 Oct 20].

[39] Odendaal WA, Anstey Watkins J, Leon N, Goudge J, Griffiths F, Tomlinson M, et al. Health workers’ perceptions and experiences of using mHealth technologies to deliver primary healthcare services: a qualitative evidence synthesis. Cochrane Database Syst Rev. 2020 Mar 26;3:CD011942. 10.1002/14651858.CD011942.pub2 32216074 PMC7098082

[40] Kavuma M. The usability of electronic medical record systems implemented in Sub-Saharan Africa: a literature review of the evidence. JMIR Human Factors. 2019 Feb 25;6(1):e9317–9317. 10.2196/humanfactors.9317 30801251 PMC6409508

[41] Kumar P, Sammut SM, Madan JJ, Bucher S, Kumar MB. Digital ^1^ paperless: novel interfaces needed to address global health challenges. BMJ Glob Health. 2021 Apr;6(4):e005780. 10.1136/bmjgh-2021-005780 33879473 PMC8061842

[42] Kaphle S, Chaturvedi S, Chaudhuri I, Krishnan R, Lesh N. Adoption and usage of mhealth technology on quality and experience of care provided by frontline workers: observations from rural India. JMIR Mhealth Uhealth. 2015 May 28;3(2):e61. 10.2196/mhealth.4047 26023001 PMC4464193

[43] Zaidi S, Kazi AM, Riaz A, Ali A, Najmi R, Jabeen R, et al. Operability, usefulness, and task-technology fit of an mhealth app for delivering primary health care services by community health workers in underserved areas of Pakistan and Afghanistan: qualitative study. J Med Internet Res. 2020 Sep 17;22(9):e18414. 10.2196/18414 32940612 PMC7530697

[44] Lippeveld T. Routine health facility and community information systems: creating an information use culture. Glob Health Sci Pract. 2017 Sep 27;5(3):338–40. https://www.ghspjournal.org/content/5/3/338 28963169 10.9745/GHSP-D-17-00319 PMC5620331

